# Evaluation of Various Surface Cleaning Techniques Inside Tunnels on Pavement Skid Resistance

**DOI:** 10.3390/ma14195660

**Published:** 2021-09-28

**Authors:** José Ramon Marcobal, Freddie Salado, Gerardo Flintsch

**Affiliations:** 1 Departamento de Ingeniería del Transporte, Territorio y Urbanismo, Technical University of Madrid, 28040 Madrid, Spain; 2 Department of Civil and Environmental Engineering, Virginia Tech Transportation Institute, 3500 Transportation Research Plaza, Blacksburg, VA 24061, USA; freds85@vt.edu; 3 Center for Sustainable Transportation Infrastructure, Virginia Tech Transportation Institute, 3500 Transportation Research Plaza, Blacksburg, VA 24061, USA; gflintsch@vtti.vt.edu

**Keywords:** Skid Resistance, hydroblasting, microgriptester, SCRIM, sweeping, water pressure

## Abstract

A request started a comprehensive investigation on the Skid Resistance (SR) inside a highway tunnel in Madrid, Spain, to determine methods that will improve or maintain the SR above the minimum required level (60), after the application of Hydroblasting, without the need of pavement surface rehabilitation. The right lane located on eight sections inside tunnel XC was used as a test section, in which three went thru the application of water pressure applied weekly, biweekly, and monthly. Three other sections for surface sweeping at the same frequencies and two untreated sections as control. Using the Microgriptester, weekly SR measurements taken before and after tracked the pavement response. Results showed that SR decreased weekly depending on the frequency of the treatment applied and for higher frequencies, SR was almost at the same level. Water pressure and sweeping applied weekly, biweekly, and monthly, during the two months testing period, maintained the SR level above the required value of 60 and the untreated sections showed that pavement surface must be treated to maintain the SR above 60.

## 1. Introduction

Most road administrators monitor wet Skid Resistance on all types of pavements due to the high probability of occurring skidding accidents [1]. According to the European Commission, more than 30,000 people died on European Union (EU) roads in 2011 due to crashes. For each death, approximately 4 people remain disabled, 8 with serious injuries and 50 with minor injuries. To reduce these deaths, the European Union adopted a Road Safety Program (RSP) that focuses on improving highway safety [2]. In 2016, the European Road Safety Observatory published the Annual Accident Report that showed the number of fatalities in European roads decreased significantly to 26,258, however, the EU failed to meet their target of a reduction of 29% by 2015 [3].

According to the report European Union Explained: Transport published in 2014, the European transport is expected to cost 1.5 trillion EUR by 2030 and 500 billion EUR will be needed by end of the RSP in 2020 to improve the transportation infrastructure, including safety and traffic congestion [4]. In Spain, the transport sector accounts for 24.5% of its economy in 2015, which proves that there is a need for countermeasures to decrease the required budget [5].

**Figure 1:**
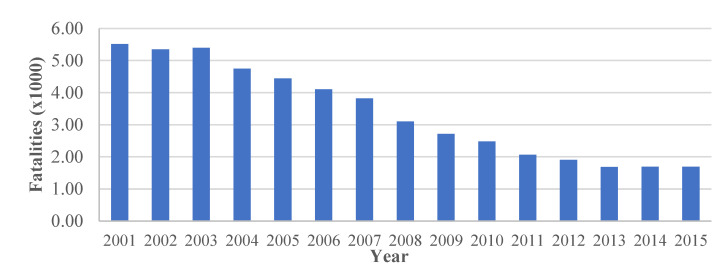
The number of fatalities in Spain between 2001 and 2015 and the EU target reduction (29%) between the end of 2010 and 2015 [6].

Road safety is a major issue in Spain, where almost 50,000 fatalities occurred between 2001 and 2015 (Figure 1). The figure shows significant reductions in fatalities in Spain since 2003, due to the efforts on improving road safety. Moreover, in 2010, the EU set a target reduction in fatalities of 29% to significantly reduce it in the short term. By the end of 2015, Spain achieved the target of reducing the fatalities, with a reduction of 32%, which represents approximately 1025 fatalities less than in 2010 than in 2015.

Although Spain achieved a reduction of 32% in fatalities very quickly after adopting its RSP, the total amount of crashes has leveled since 2013. Pavement surface studies on highways located in Madrid, Spain, suggested that pavement surface properties were not consistently adequate for the road user. The study found a lack of friction in many locations, especially inside tunnels where the maximum speed is approximately 45 mph (70 km/h), which increases the probability of crashes. The World Road Association found an average of 331 crashes per year in the tunnel system along a highway in Madrid [7]. This prompted a comprehensive investigation of the Skid Resistance (SR) inside tunnels to determine methods to improve the SR on pavement surface without the need for pavement surface rehabilitation.

### 1.1. Objective

The objective of the study was to evaluate the feasibility of two cleaning techniques, namely, water pressure and sweeping, applied weekly, biweekly, and monthly to extend the effectiveness of the hydroblasting procedure conducted on pavement inside a tunnel.

### 1.2. Project Background

Research studies conducted optimized the application procedures of chemical agents that eliminate the residual particles on the tunnel’s pavement. The first technical note, issued in November 2010, presented the analysis conducted, the technical requisites related to the SR, and the work performed by a contractor for the evaluation of different techniques to improve the SR [8].

Section 2.3.1 of the Royal Decree 635/2006 [9], related to the minimum safety requirements in state road tunnels, states a minimum SR of 60. The Sideway-force Coefficient Routine Investigation Machine (SCRIM) permits the measurement of the “Coeficiente de Rozamiento Transversal (CRT)” (Transversal Friction Coefficient), which is a measurement of SR [10]. From the study conducted, the SR was above the required level in most of the pavement surface; however, several segments inside tunnels were below the required level, suggesting that there was a need for further investigation.

As part of the effort to improve the SR inside tunnels, the contractor decided to apply hydroblasting on the pavement surface. Hydroblasting has been used for pavement marking removal, runway rubber removal, curing compound removal, surface preparation, bridge cleaning, and pavement surface cleaning to remove the debris accumulated due to the traffic and weather [11,12]. The removal of the debris can restore Skid Resistance and texture to specified levels by exposing the microtexture at a low cost while preventing damaging the pavement surface and the risk of crashes due to hydroplaning [13].

The hydroblasting on the pavement surface inside the tunnel was very effective as it increased the average SR from 46 to 71; however, posterior measurement showed that the SR decreased to an average SR of 57 after only a year. Hydroblasting proved to be an effective method to restore SR compared to rehabilitation methods such as cold milling when it comes to cost, application time, and the impact on the road user. However, its effect did not last long, prompting the study presented in this paper to explore methods to slow the friction degradations. The study focused on how to maintain the SR above the required levels without the need of applying hydroblasting frequently.

## 2. Research Methodology

The research plan included two phases, a laboratory study and a field study consisting of periodic measurement at the pavement sections inside the tunnel. For the laboratory phase, samples were taken from the pavement inside the tunnel and the material that accumulates on the pavement surface to test its characteristics as a lubricant and abrasive. The second phase compared the evolution of SR with the application of periodic water pressure and sweeping treatments at three frequencies. This paper presents the analysis conducted in the second phase of the study.

### 2.1. Laboratory Study

The lubricant and abrasive properties of the material that accumulates on the pavement surface were analyzed in this phase. Samples were taken from the ventilation filters inside the XC tunnel and of the coarse aggregate of the pavement surface. Following the standard UNE-EN 1097-8 [14], the effect of the lubricant was studied by comparing the Skid Resistance of a sample tested, using the British Pendulum, which contains the material taken from the ventilation filters with that of a sample without the material [14]. The abrasive effect of the material was tested by comparing the Polish Stone Value (PSV) of the samples with different abrasives, including the one collected from the filters.

Results from both tests showed that the particles accumulated on the pavement surface inside the tunnel played a significant role in the Skid Resistance reduction after the application of hydroblasting. This suggested that cleaning technics that do not have a major impact on the traffic would help maintain high SR of the pavement surface.

### 2.2. Field Study

Two cleaning techniques were studied during this phase to verify the effect of the treatments on reducing the SR degradation after the application of hydroblasting. The contractor tested several cleaning techniques in previous studies and selected two techniques for this study due to the effectiveness and easiness of application. The treatments were applied at three frequencies: weekly, biweekly, and monthly. The first treatment, water pressure washing, consisted of applying water on the pavement surface of each lane at approximately 3600 psi with a flow rate of 215 L/min, to wash the particles to the drainage systems. The second treatment consisted of sweeping the pavement surface with a mechanical brush at 100 rpm to remove the solid particle from the surface of each lane.

## 3. Antecedents and Experimental Design

Sections were selected along one tunnel (XC) to reduce a possible variability of the results within sections. Selecting the sections from the same tunnel helps minimize variations in traffic volume, pavement structure, and vehicle classification. The following criteria were used to select the sections:

- Traffic: Acceleration and deceleration lanes were not included due to the variability of the SR values in the same location caused by the constant braking of the vehicles. Sections with almost constant volume and speed were selected;
- Length: Each section has a minimum length of 200–250 m;
- Lane location: Due to the high traffic volume inside the tunnel, only the right lane was tested to minimize the impact on the traffic;
- Referencing: Kilometer markers, or kiloposts, were used to indicate the begging and end of each section inside the tunnel;
- Pavement type: All sections selected have the same pavement type.

### 3.1. Sections Selected

The eight selected sections are located on the right lane of the XC tunnel, and each has an approximate length of 250 m.

Table 1 shows the location of the start and end of each studied section inside the tunnel.

Since the tunnel only has one entrance and one exit, the traffic volume shown in Table 2 can be considered uniform along all the pavement sections. Traffic counts conducted during the testing period showed an average 4.5% traffic increase every month, which was not considered a substantial increment that can affect the treatment application during the 2 months.

The pavement surface is homogenous along the tunnel as the pavement inside the tunnel was paved in 2007 using a gap-graded mixture type BBTM-B, which covered all the sections tested in this study.

### 3.2. Treatment Distribution

As previously mentioned, two low-cost treatments, water pressure and sweeping, were studied in terms of their frequency of application. Two sections located in the middle of the tunnel were used as control and received no treatment during the study. The other six sections were divided into two groups of three sections to test Hydroblasting and Sweeping at different frequencies.

Table 3 below shows a matrix of the treatment distribution and frequency among the eight sections. From the kilopost 12XC00 until 11XC25, three sections have been subjected to water pressure on the surface every week, every two weeks, and every month for two months. The contractor performed the second treatment, sweeping, on the pavement surface of the last three sections, from the kilopost 10XC75 until 10XC00, every week, every two weeks, and at the end of each month during the two months of the study.

Although each section has a total length of 250 m, considering the distance between each kilopost, the total distance of the measured SR ranges between 200 and 250 m.

## 4. Results

The data analysis conducted for each treatment response shows if the SR increased significantly after the application of the treatment and at what frequency, or if the SR maintained its value during the two weeks. Before studying the individual and general response of the treatments applied on the pavement surface, the data acquired from the Microgriptester for every week required filtering to find outliers. Results from the filtering process concluded that the data taken from every section under study required a 5% removal of outliers. After arranging the data set in increasing order, the analysis conducted did not include 5% of the lower and top parts considered as outliers.

### 4.1. Treatments General Response

The data collected every 5 cm with the Microgriptester showed discrepancies such as increment of SR without the application of a treatment or for the case of the control sections, SR increased and decreased from week to week. To reduce the risk of having errors in the response of the treated and untreated sections, a reduction of 5% of the outliers from the bottom and the top of the data set made possible a better and more accurate view of the results. Table 4 shows the data considered in the study, including the quantity of data analyzed and the mean SR for each. Only the two untreated (control) sections considered the measured SR during the first week and the last week of the analysis and for the rest of the sections the included mean SR depended on the frequency of application.

**Figure 2:**
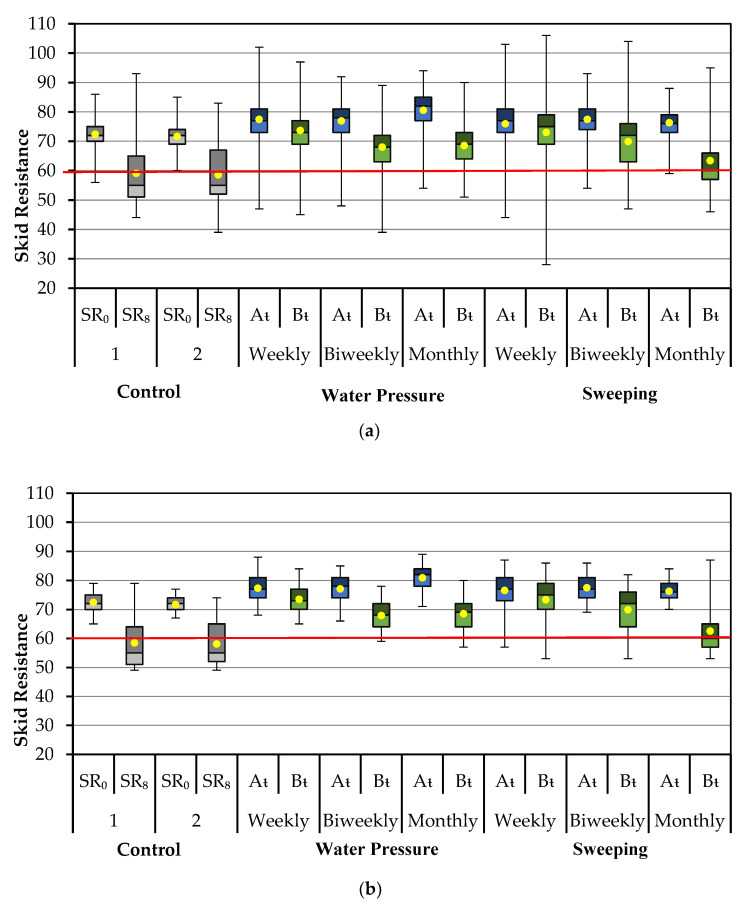
Box plot and whiskers of SR with (**a**) and without 5% of the outliers (**b**).

The calculated mean SR for each section with and without outliers did not change more than 1% compared to the original mean SR, which allows us to have certainty that the outliers removed did not represent a major change in the average response of the treatment applied. Although the mean SR did not change significantly after the outlier removal, Figure 2a,b show a different perspective of the impact of removing 5% of those outliers from each tested section.

The box plot and whiskers are shown in Figure 2a, which includes the entire SR data set. The first two sets show the SR measured on the untreated sections during the first day (SR_0_) and at the end of the study during the eighth week (SR_8_). The following sets present the SR measured at treated sections right before the applied treatment (B_t_) and after the applied treatment (A_t_). Thus, for the section with water pressure applied weekly, A_t_ represents SR measured immediately after the treatment and B_t_ represents the SR measured a week later, just before the next treatment application. Although the whiskers plotted from the data acquired, from each section, show high variability, the plot shows the decreasing trend for each treatment and frequency. Both plots have the same scale on the y axle, which helps to visualize how the whiskers reduced drastically and are closer to the mean value after removing 5% of the outliers (Figure 2b).

Figure 2b shows the unacceptable average SR from the control sections after two months, which proves the need for treatment. At the end of the second month, 63.8% and 68.2% of the SR measured at each untreated section were below 60.

Sections with water pressure applied weekly showed a good response in which 100% of the SR measurements maintained the SR above 60. Approximately 2.3% and 6.1% of SR measurements fall below 60 for the biweekly and monthly frequency, respectively.

SR measurements taken from sweeping weekly and biweekly were mostly above 60, and only 8.1% and 16%, respectively, were below. Although sweeping monthly achieved a higher mean SR compared to the other frequencies and treatment, 46.9% of the SR measurements were below 60 a month after the treatment.

Both treatments allow maintaining the average SR above 60, proving effectiveness at every frequency applied. For a cost-effective solution, the monthly application of water pressure maintains the average SR above the minimum limit. However, for the sweeping of the pavement surface, a monthly frequency will not prevent the SR from being below the required level for a short period. Sweeping biweekly will bring the needed performance to maintain the SR above the required level.

### 4.2. Treatments Effect

A one-tailed *t*-test determines if there is statistically significant evidence at α = 0.05, to show that the mean of the SR measurements (*µ_SR_*) taken just before and after the treatment is more than 60. The null hypothesis states that *µ_SR_* is equal to 60 and the alternate that the *µ_SR_* is greater than 60.

(1) $$\begin{array}{c} H_{0}:{µ}_{SR}=60\\ H_{a}:{µ}_{SR}>60 \end{array}$$

If the calculated *t*-test is greater than t_α_, we can conclude that the mean SR measured is more than 60 with statistically significant evidence at α = 0.05.

### 4.3. Water Pressure

Table 5 presents the results obtained from the one-tailed *t*-test analysis conducted for the before and after treatment SR. Measurements were taken every week for each section; however, the analysis only considered the measure taken immediately before and after the treatment. For example, for a monthly frequency, the analysis included only the measurements taken at the end of each month. After the application of hydroblasting, the mean SR obtained from each section was considered as baseline (Mean_BL_).

Results show that the null hypothesis is rejected since the t-statistic > t_0.05_. There is statistically significant evidence at α = 0.05 that the mean SR measured before and after the treatment is greater than 60. Since the Mean_BL_ for each section is the same, a comparison can be made between frequencies. The frequency of treatment application shows a decreasing trend, in which the higher the frequency, the higher the mean SR. Table 5 also shows that even though the section was not treated for an entire month, it still maintains the SR above 60.

### 4.4. Sweeping

Following the same procedure stated for water pressure, Table 6 presents the results obtained from the one-tailed *t*-test. The null hypothesis is rejected, therefore there is statistically significant evidence at α = 0.05 that the mean SR measured before and after the treatment is greater than 60. As a general observation, the mean SR for each section shows that for lower frequencies, the SR increases. Considering analyzing each section, unlike water pressure, the sections used for sweeping have different Mean_BL_. This means that comparisons are possible between weekly and biweekly because the section with a monthly frequency started with a Mean_BL_ = 76.

The decreasing trend from the before treatment measurements proves that for lower frequencies, the SR reduces drastically, making it closer to 60. The after-treatment results suggest that sweeping the pavement surface frequently (weekly) does not maintain the SR higher than monthly. However, by comparing the Mean_BL_ of each section with the calculated mean, a monthly frequency maintained the SR 4 friction numbers higher, while weekly and biweekly maintained the SR approximately 10 friction numbers higher.

Both treatments increased the average SR above 72 for every frequency. However, it does not prove that water pressure applied weekly is the feasible solution to maintain SR above 60 after the application of hydroblasting. When it comes to minimizing the impact to the traffic and finding an economic treatment application, water pressure applied monthly proves to be effective at maintaining SR above 60.

Every treated section maintained the SR value above the minimum required but sweeping applied monthly presented a better performance. A possible explanation could be due to the difference in Mean_BL_ obtained after the hydroblasting application. The section with a monthly frequency started with an SR = 76, which was 10 friction numbers higher than the weekly and biweekly frequencies. The section performance was the best by increasing the SR after treatment, but when it comes to maintaining the SR after a month, 50% were below 60.

## 5. Findings

According to the study conducted on the application performance of water pressure and sweeping applied on eight sections located inside tunnel XC in Madrid, Spain, the following findings emerged:

- The 5% outliers removal from the bottom and the top did not change more than 1% of the average SR compared to the original;
- Outliers removal helped with the reduction of unrelated data that does not present the actual performance of the tested sections;
- Box plots showed how SR decreased depending on the frequency of the treatment applied;
- Both treatments applied maintained the SR above 60 at every frequency;
- Water pressure applied monthly proves to be efficient in maintaining SR above the minimum level without major impacts to the traffic and pavement surface;
- Pavement surface sweeping can also increase the SR but at a slower rate compared to the water pressure;
- The average weekly performance of the sections proved that for higher frequencies, SR maintained its value above 60;
- SR of the control sections suggests that surface treatment is needed to maintain the SR above 60;
- One-tailed *t*-test showed that for both treatments and frequencies, there is statistically significant evidence at α = 0.05 that the SR is greater than 60 after it is applied.

## 6. Conclusions

In summary, after the application of hydroblasting, water pressure, and sweeping prove to be effective. The treatments applied weekly, biweekly, and monthly, during the two months testing period, maintained the SR level above the required value of 60. The untreated sections showed that pavement surface must be treated to maintain the SR above 60.

## 7. Recommendations

After concluding the analysis of the application of the treatments, the following recommendations bring the possibility for future research on this topic:

- The testing period of two months does not give an entire view of pavement response in terms of when it will reach the minimum value of 60, therefore frequencies of two months or more will help to find that threshold;
- Georeferenced SR measurements before and after treatment to avoid outliers;
- Analysis of the economic impact of the treatment;
- Addition of other treatments such as air blowing or vacuum and shot blasting.

## Figures and Tables

**Table 1:** Location of studied sections inside tunnel XC.

**Section**	**1**	**2**	**3**	**4**	**5**	**6**	**7**	**8**
Start	12XC00	11XC75	11XC50	11XC25	11XC00	10XC75	10XC50	10XC25
End	11XC75	11XC50	11XC25	11XC00	10XC75	10XC50	10XC25	10XC00

**Table 2:** Volume inside tunnel XC.

**Month**	**Volume(Veh./Day)**	**Light Vehicle Volume (Veh./Day)**	**Heavy Vehicle Volume (Veh./Day)**
April	9754	7998	1756
May	10,440	8039	2401
June	10,750	8600	2150

**Table 3:** Treatment distribution and frequency for each section.

**Section**	**1**	**2**	**3**	**4**	**5**	**6**	**7**	**8**
Start	12XC00	11XC75	11XC50	11XC25	11XC00	10XC75	10XC50	10XC25
End	11XC75	11XC50	11XC25	11XC00	10XC75	10XC50	10XC25	10XC00
Treatment	Water Pressure			No Treatment		Sweeping		
Frequency	Weekly	Biweekly	Monthly			Weekly	Biweekly	Monthly

**Table 4:** Count and mean SR of the data set and without 5% of outliers.

Treatment	Frequency		0% of Outliers Removed		5% of Outliers Removed	
			Count	Mean	Count	Mean
Untreated	N/A	SR_0_	3862	72.4	3476	72.4
		SR_8_		59.1		58.4
	N/A	SR_0_	3903	71.6	3513	71.6
		SR_8_		58.5		58.1
Water Pressure	Weekly	A_t_	15,859	77.4	14,273	77.3
		B_t_		73.6		73.4
	Biweekly	A_t_	8057	76.9	7251	77.0
		B_t_		67.9		67.8
	Monthly	A_t_	3880	80.5	3492	80.9
		B_t_		68.5		68.5
Sweeping	Weekly	A_t_	28,631	75.9	25,767	76.5
		B_t_		73.0		73.3
	Biweekly	A_t_	8535	77.4	7681	77.4
		B_t_		69.9		69.9
	Monthly	A_t_	4064	76.3	3658	76.2
		B_t_		63.4		62.5

**Table 5:** One-tailed *t*-test of SR measurement-water pressure.

	Frequency	Count	Mean_BL_	Mean	SD	t_0.05_	T-Statistic	*p*-Value
Before	Weekly	20,414	73.0	74.48	6.33	1.64	326.84	0.000
	Biweekly	13,657	73.0	69.5	6.2	1.64	177.98	0.000
	Monthly	7872	73.0	67.77	6.5	1.64	104.61	0.000
After	Weekly	20,414	73.0	78.1	6.5	1.64	393.62	0.000
	Biweekly	13,657	73.0	75.5	6.4	1.64	283.02	0.000
	Monthly	7872	73.0	72.1	11.	1.64	96.33	0.000

**Table 6:** One-tailed *t*-test of SR measurement-sweeping.

	Frequency	Count	Mean_BL_	Mean	SD	t_0.05_	T-Statistic	*p*-Value
Before	Weekly	32,047	66.0	72.6	9.44	1.64	240.27	0.000
	Biweekly	12,743	66.0	70.6	8.6	1.64	139.07	0.000
	Monthly	5530	76.0	65.86	9.52	1.64	45.77	0.000
After	Weekly	32,047	66.0	75.2	9.3	1.64	292.58	0.000
	Biweekly	12,743	66.0	77.2	7.8	1.64	250.08	0.000
	Monthly	5530	76.0	80.1	10.5	1.64	142.84	0.000

## Data Availability

Data sharing not applicable.

## References

[1] Wilcox R.G. (1973). Skid Resistance of Highway Pavements.

[2] European Commission Statistics—Accidents Data. Mobility and Transport: Road Safety. https://ec.europa.eu/transport/road_safety/specialist/statistics_en.

[3] European Commission Annual Accident Report 2016. Mobility and Transport: Road Safety. https://ec.europa.eu/transport/road_safety/sites/roadsafety/files/pdf/statistics/dacota/asr2016.pdf.

[4] European Union Countries Europa. 10 May 2017. https://europa.eu/european-union/about-eu/countries_en.

[5] European Union Spain: Overview. Europa. 26 June 2017. https://europa.eu/european-union/about-eu/countries/member-countries/spain_en.

[6] European Commission European Road Fatalities. Directorate General for Mobility and Transport. https://ec.europa.eu/transport/road_safety/sites/roadsafety/files/pdf/observatory/trends_figures.pdf.

[7] World Road Association Modiale De La Route PIARC WG5 “Complex Underground Road Networks”—Part A Case Studies. Appendix 2.17-SPAIN–Madrid-Riío-TUNNEL. https://tunnels.piarc.org/en/system/files/media/file/appendix_2.17_-_spain_-_madrid_-_rio_tunnel.pdf.

[8] ESTI de Canales, Caminos y Puertos (2011). Convenio Para la Optimización de los Procedimientos de Aplicación de Agentes Químicos para la Eliminación de la Contaminación en los Pavimentos de Túneles: Diseño Experimental.

[9] Ministerio de Fomento Real Decreto 635/2006, Sobre Requisitos Mínimos de Seguridad en los Túneles de Carreteras del Estado. Agencia Estatal: Boletín Oficial del Estado. https://www.boe.es/diario_boe/txt.php?id=BOE-A-2006-9296.

[10] Euroconsult Nuevas Tecnologías SCRIM (Control de Adherencia Sobre Pavimentos Mojados). http://www.euroconsult.es/pdf/Scrim_multiidioma.pdf.

[11] Zimmerman Paint Contractors, Co The Advantages of Hydroblasting for Line Removal and Surface Preparation. http://zimmermanpaint.com/html/line-removal-hydroblasting.html.

[12] Performance Hydroblasting Road Marking Removal. Performance Hydroblasting Professional Services. http://www.performancehydroblasting.com/roadmarkingremoval.

[13] H2-GO The Benefits and Advantages of Using H2-GO’s Hydroblasting Services. Why H2-Go?. http://www.h2-go.com/page/the-benefits-and-advantages-of-using-h2-gos-hydroblasting-services/.

[14] UNE-EN 1097-8 (2010). Ensayos Para Determinar las Propiedades MECÁNICAS y Físicas de los Áridos. Depósito Legal: M 1223:2010.

